# Seroprevalence of Binding and Neutralizing Antibodies against 39 Human Adenovirus Types in Patients with Neuromuscular Disorders

**DOI:** 10.3390/v15010079

**Published:** 2022-12-27

**Authors:** Patrick Julian Klann, Xiaoyan Wang, Anna Elfert, Wenli Zhang, Cornelia Köhler, Anne-Katrin Güttsches, Frank Jacobsen, Ute Weyen, Andreas Roos, Eric Ehrke-Schulz, Anja Ehrhardt, Matthias Vorgerd, Wibke Bayer

**Affiliations:** 1 Institute for Virology, University Hospital Essen, University Duisburg-Essen, 45122 Essen, Germany; 2 Heimer Institute for Muscle Research, Department of Neurology, University Hospital Bergmannsheil, Ruhr-University Bochum, 44789 Bochum, Germany; 3 Virology and Microbiology, Center for Medical Education and Research, Department of Human Medicine, Faculty of Health, Witten/Herdecke University, 58453 Witten, Germany; 4 Clinics for Pediatrics and Adolescent Medicine, University Hospital Sankt Josef, Ruhr-University Bochum, 44791 Bochum, Germany

**Keywords:** human adenovirus, HAdV, viral vector, gene therapy, neuromuscular disease, seroprevalence, neutralizing antibodies, binding antibodies

## Abstract

High pre-existing antibodies against viral vectors reduce their functionality and may lead to adverse complications. To circumvent this problem in future gene therapy approaches, we tested the seroprevalence of a large range of human adenovirus types in patients with neuromuscular disorders (NMDs) to find appropriate viral vector candidates for gene replacement therapy for NMDs. Binding and neutralizing antibodies against 39 human adenovirus types were tested in the sera of 133 patients with NMDs and 76 healthy controls aged 17–92 years. The influence of age, sex, and NMDs on antibody levels was analyzed. The seroprevalence of different adenoviruses in the cohort varied widely. The highest levels of binding antibodies were detected against HAdV-D27, -C1, -D24, -D70, -B14, -C6, -D13, -B34, and -E4, whereas the lowest reactivity was detected against HAdV-F41, -A31, -B11, -D75, -D8, -D65, -D26, -D80, and -D17. The highest neutralizing reactivity was observed against HAdV-B3, -C2, -E4, -C1, -G52, -C5, and -F41, whereas the lowest neutralizing reactivity was observed against HAdV-D74, -B34, -D73, -B37, -D48, -D13, -D75, -D8, -B35, and -B16. We detected no influence of sex and only minor differences between different age groups. Importantly, there were no significant differences between healthy controls and patients with NMDs. Our data show that patients with NMDs have very similar levels of binding and neutralizing antibodies against HAdV compared to healthy individuals, and we identified HAdV-A31, -B16, -B34, -B35, -D8, -D37, -D48, -D73, -D74, -D75, and -D80 as promising candidates for future vector development due to their low binding and neutralizing antibody prevalence.

## 1. Introduction

Adenoviruses are non-enveloped, icosahedral, double-stranded DNA viruses belonging to the family of Adenoviridae and have been studied as viral vectors for immunization and gene therapy and as oncolytic agents for a long time. Currently, there are 113 human adenovirus (HAdV) types recognized by the scientific community [1] grouped into the seven species A through G [2] and over 200 non-human types, leading to a variety of opportunities for the future design of viral vectors in biomedicine [3]. High pre-existing antibodies against the HAdV types that were initially used for vector development, species C human adenovirus type 2 (HAdV-C2) and HAdV-C5 [4,5,6,7,8], can result in impaired transgene delivery efficacy [9], and the induction of immune responses was found to be reduced in individuals with high pre-existing immunity in pre-clinical trials of HAdV-C5 based vaccines [10,11]. Therefore, new strategies have focused on the utilization of different virus subtypes with lower seroprevalence for vector development [12]. Important advances have since been made in the use of HAdV vectors especially in the field of vaccine development, and have led to the licensing of HAdV based vaccines against Ebola virus [13,14] and SARS-CoV-2 [15,16]. While first-generation vectors with E1 and E3 genes deleted are commonly used for vaccine vector development, their high immunogenicity is a major drawback for their application in gene therapy, and high-capacity vectors that are devoid of any AdV-derived genes are now the favored candidates for gene therapy applications [3].

Neuromuscular disorders (NMDs) include a large variety of different inherited and acquired subtypes affecting different cellular populations across the neuromuscular axis (motoneuron–peripheral nerves–neuromuscular junction–muscle cells). Accordingly, these disorders can be caused by functional defects manifesting in anterior horn cells, peripheral nerves (Schwann cells and axons), the neuromuscular junction (pre-synaptic, synaptic and post-synaptic), and skeletal muscle cells or might even affect more than one of these, with neuromyopathies being prominent examples [17]. In many cases, the first symptoms manifest in childhood and often comprise myalgias, muscle weakness, numbness, spasms and sensory symptoms. These disorders are often progressive with increasing age and may lead to severe disability, including severe to complete paralysis, and possibly death through respiratory or cardiac dysfunction [18]. Many NMDs are monogenic disorders, making them promising candidates for treatment by gene therapy. Indeed, one gene replacement therapy has been successfully developed for the treatment of spinal muscular atrophy (5q-SMA). This gene therapy agent is based on adeno-associated virus serotype 9 (AAV9) [19] and has been approved as onasemnogene abeparvovec (Zolgensma) by the U.S. Food and Drug Administration in 2019 and by the European Medicine Agency in 2020 [20]. 

More gene replacement therapies for NMDs based on AAVs are currently tested in clinical trials and pre-clinical animal models (see [21,22] for reviews) for the treatment of Duchenne muscular dystrophy [23,24], amyotrophic lateral sclerosis (ALS) [25], alpha-sarcoglycan deficiency [26], Pompe’s disease [27], Charcot-Marie-Tooth disease (CMT) [28], or familial limb-girdle myasthenia [29]. AAVs have many advantages as viral vectors based on their replication deficiency, their ability to transduce non-dividing tissues and their low potential for pathogenicity. Nevertheless, they present with the major disadvantage of low carrying capacity: the genome size of AAVs is only 4.8 kb and the open reading frames of some genes are by far too long, with the *DMD* gene which contains 2.5 Mb of the genomic DNA forming a 14 kb mRNA transcript [30], being a striking example of a target sequence that cannot be carried by AAVs [31]. In contrast to AAVs, the coding capacity of HAdV ranges between 8.5 kb and 36 kb depending on the vector design [32]. Accordingly, also HAdV based vectors have been used in experimental gene therapy studies in preclinical models of ALS [33,34,35], Pompe’s disease [36,37,38,39,40], and Duchenne muscular dystrophy [41,42,43,44,45,46,47,48,49].

**Figure 1:**
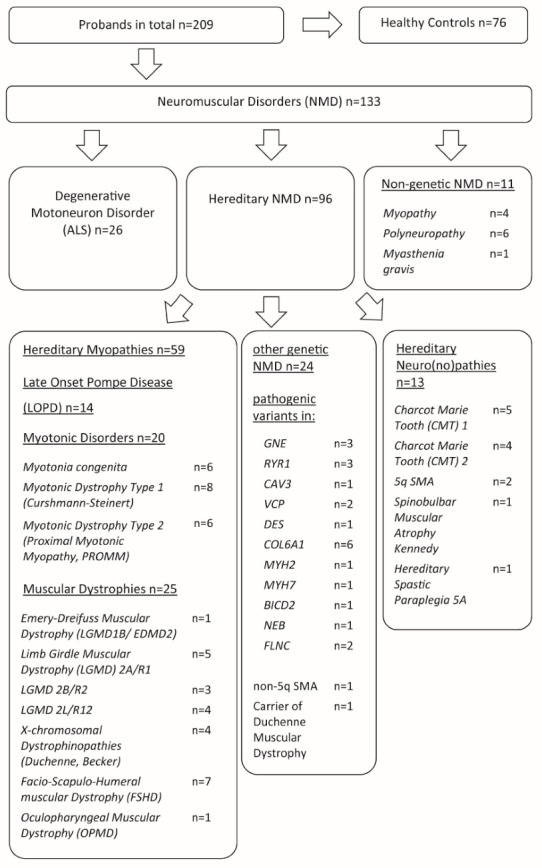
Serum donor cohort. Sera were collected from 209 patients, 133 of the serum donors were patients with NMD, and 76 were healthy controls without NMD. Patients with NMD comprised patients with degenerative motoneuron disorder, non-genetic NMD and hereditary NMD, including hereditary myopathies, hereditary neuro(no)pathies and other genetic NMD.

The above-mentioned preclinical trials for NMD gene therapy have used HAdV-C5 based vectors. However, recent years have brought many efforts to develop novel HAdV vectors based on so-called rare HAdV types to allow more effective gene delivery by circumventing pre-existing immunity. In recent years, many studies have been performed to analyze the seroprevalence of different HAdV types in different healthy populations (reviewed in [50,51]). In many cases, the studies focused on only a small number of HAdV types, with the notable exceptions of a study of neutralizing antibodies against 33 HAdV types in young children in Italy [52] and the study of neutralizing antibodies against 51 HAdV types in a Belgian blood donor cohort [53]. We have ourselves recently performed an analysis of binding and neutralizing antibodies against 39 HAdV types in a cohort of young healthy adults [54], where we could identify a number of low-prevalent HAdV types mainly from species A, B, and D that may be promising candidates for future vaccine and gene therapy vector development. To expand on these findings, in the study presented here, we investigated the seroprevalence in patients suffering from different NMDs. Here, we exclusively focused on degenerative neuromuscular entities in adult patients (Figure 1), which may be target diseases for gene replacement therapy approaches in the future [55].

## 2. Materials and Methods

### 2.1. Ethics Approval

The present study was reviewed and approved by the Ethics Committee of the Medical Faculty of the Ruhr-University Bochum (20–6924). All serum donors provided their written informed consent to participate in this study, and to the use of the data for publication.

### 2.2. Human Samples

A total of 209 serum samples were collected from adult patients of the neuromuscular ambulance of the University Hospital Bergmannsheil (Heimer Institute for Muscle Research; Ruhr University, Bochum, Germany) between August 2020 and March 2021: 76 from healthy adult volunteers and 133 from patients with different NMDs. Their ages ranged from 17 to 92 years. Patients with NMDs were subdivided into 7 groups: 14 patients with late-onset Pompe disease (LOPD), 25 patients with muscular dystrophies, 26 patients with degenerative motoneuron disease (ALS), 20 patients with myotonic disorders, 13 patients with hereditary neuro(no)pathies, 24 patients with other defined genetic NMDs and 11 patients with other non-genetic NMDs (Figure 1, Table 1). Inclusion criteria consisted of written consent to participate in the study and a reliable molecular genetic and clinical diagnosis of an NMD. Clinical diagnoses were made by consultants specialized in neuromuscular diseases. Exclusion criteria were an inflammatory myositis, an immunosuppressive therapy, known infections with human immunodeficiency virus or hepatitis B or C virus, or the missing ability to consent. All samples were centrifuged for 10 min and the sera were stored at –80 °C.

### 2.3. Cells

The human lung carcinoma cell line A549 (ATCC CCL-185, LGC Standards, Wesel, Germany) and the human embryonic kidney cell line 293A (Microbix Biosystems, Toronto, ON, Canada) [56] were used in this study. Both cell lines were cultured in Dulbecco’s modified Eagle’s medium (DMEM) supplemented with 10% heat inactivated fetal calf serum (FCS), 50 mg/L gentamycin (all cell culture reagents were obtained from Invitrogen/Gibco, ThermoFisher Scientific, Karlsruhe, Germany), and 20 mg/L ciprofloxacin (Fresenius Kabi, Linz, Austria). The cells were cultured at 37 °C in a 5% CO_2_ atmosphere.

### 2.4. Viruses

The HAdV types used in this study have been described in detail previously [54]. Briefly, we used 39 HAdV types: HAdV-A12, -A18, -A31, -B3, -B7, -B11, -B14, -B16, -B21, -B34, -B35, -B50, -C1, -C2, -C5, -C6, -D8, -D9, -D10, -D13, -D17, -D20, -D24, -D25, -D26, -D27, -D33, -D37, -D48, -D65, -D69, -D70, -D73, -D74, -D75, -D80, -E4, -F41, and -G52. Most HAdV were wild-type viruses, except for HAdV-B16, -B50, -D48, -D65, and -D69, where we used GFP and/or luciferase encoding vectors as described previously [54]. All HAdV types were purified by a CaptoCore (Sigma-Aldrich, Taufkirchen, Germany) bead-based method adapted from [57], cesium chloride gradient ultracentrifugation or AdenoPack20 kit (Vivascience, Hannover, Germany). As reported before, the different purification methods did not influence the binding antibody ELISA results [54].

### 2.5. Binding Antibody ELISA

The binding antibody ELISA was performed as described in detail previously [54]. Briefly, 384-well Nunc Maxisorp plates (Sigma Aldrich, Taufkirchen, Germany) were coated with 2.5 × 10^7^ viral particles (vp) per well of purified, UV-inactivated (254 nm, 30 min, 4 °C) HAdV in PBS overnight at 4 °C. After washing with PBS + 0.1% Tween20 (PBS-T) and blocking with PBS + 20% fetal calf serum, 20 μL of serum samples diluted 1:1000 in PBS were added to the wells prior to incubation at 4 °C overnight. Binding antibodies were detected using a horseradish peroxidase labelled polyclonal donkey-anti-human IgG antibody (Dianova, Hamburg, Germany) and tetramethylbenzidine as substrate (1-Step TMB Ultra, ThermoFisher, Waltham, MA, USA). Absorption at 450 nm was analysed using a Mithras^2^ microplate reader (Berthold Technologies, Bad Wildbad, Germany) after the addition of 25 μL 1N H2SO4.

### 2.6. IC ELISA for Detection of Neutralizing Antibodies

The analysis of neutralizing antibodies was performed using an intracellular ELISA for the detection of infected cells as described in detail previously [54]. Briefly, serial 5-fold dilutions of sera in DMEM ranging from 1:10 to 1:31, 250 (25 µL) were mixed with 10^6^ vp/25 µL of the different HAdV types 96 well flat bottom plates. Control reactions with 10^6^ vp and 10^5^ vp HAdV without addition of human serum were carried along as five replicates each on each individual plate. After incubation at 37 °C for 60 min, 1 × 10^4^ 293A or A549 cells were added to the plates and incubated for 26–138 h as described previously. For HAdV detection, the cells were fixed with PFA, permeabilized with 1% Ecosurf (ThermoFisher Scientific, Waltham, MA, USA) in PBS, and blocked with 10% FCS in PBS. Detection of HAdV hexon was performed using anti-adenovirus hexon antibody 8C4 (BioConnect, Huissen, The Netherlands), a horseradish peroxidase-labelled rabbit anti-mouse antibody (Dako Agilent, Santa Clara, CA, USA) and 1-Step TMB Ultra substrate. Plasma dilutions were considered neutralizing when the absorption values obtained for the dilution were lower than those for the 10^5^ vp control, i.e., when more than 90% of the input virus was neutralized.

### 2.7. Statistical Analysis

Statistical analysis was performed in GraphPad Prism 8 software (GraphPad Software, La Jolla, CA, USA), using Kruskal–Wallis one-way ANOVA on ranks and Dunn´s multiple comparisons test or the Chi square test with Bonferroni adjustment for multiple comparisons. 

## 3. Results

To investigate the seroprevalence against different HAdV types in adult patients with neuromuscular disorders (NMDs), we obtained samples derived from 133 patients with an NMD (Figure 1, Table 1). Of those patients, 14 presented with LOPD, 25 had a muscular dystrophy, 26 had ALS, 20 had a myotonic disorder, 13 had a hereditary neuro(no)pathy, 24 had defined genetic NMD, and 11 had other non-genetic NMD. Another 76 serum donors without NMDs were included in the study serving as a healthy control group. Of all serum donors, 102 were men and 107 were women: in the disease group 71 were men and 62 were women, in the healthy control group 31 were men and 45 were women. The mean age of the healthy control and the NMD groups was different, with a mean age of 60.12 (standard deviation (SD): 20.68) and 52.12 (SD: 15.76), respectively.

**Figure 2:**
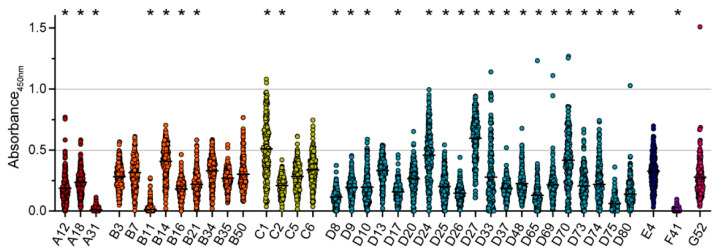
Binding antibody levels. Binding antibodies were analyzed using 209 samples of healthy volunteers and volunteers with NMD. Serum samples were diluted 1:1000 and tested for their reactivity against the 39 indicated HAdV types. Absorbance levels at 450 nm for each serum sample for the indicated HAdV types are shown. Each dot represents an individual sample, lines indicate mean values of all sera for the indicated HAdV type. All samples were tested side-by-side on the same plate for each HAdV type. Significant differences compared to HAdV-C5 are indicated by * (*p* < 0.05, Kruskal-Wallis one-way ANOVA on ranks, Dunn´s multiple comparisons test).

When we analyzed the binding antibody levels against 39 different HAdV types, we found a widely differing reactivity between HAdV types (Figure 2). The highest levels of binding antibodies were detected against HAdV-D27, HAdV-C1, HAdV-D24, HAdV-D70, HAdV-B14, HAdV-C6, HAdV-D13, HAdV-B34, and HAdV-E4, in descending order. The lowest reactivity was detected for HAdV-F41, HAdV-A31, HAdV-B11, HAdV-D75, HAdV-D8, HAdV-D65, HAdV-D26, HAdV-D80, and HAdV-D17, in ascending order. Compared to the serum reactivity against HAdV-C5, which is generally considered a highly prevalent HAdV type, the serum reactivity against HAdV-B14, HAdV-C1, HAdV-D24, HAdV-D27 and HAdV-D70 was significantly higher. On the other hand, serum reactivity against HAdV-A12, -A18, and -A31, HAdV-B11, -B16, and -B21, many species D types, and HAdV-F41 was significantly lower compared to HAdV-C5.

**Figure 3:**
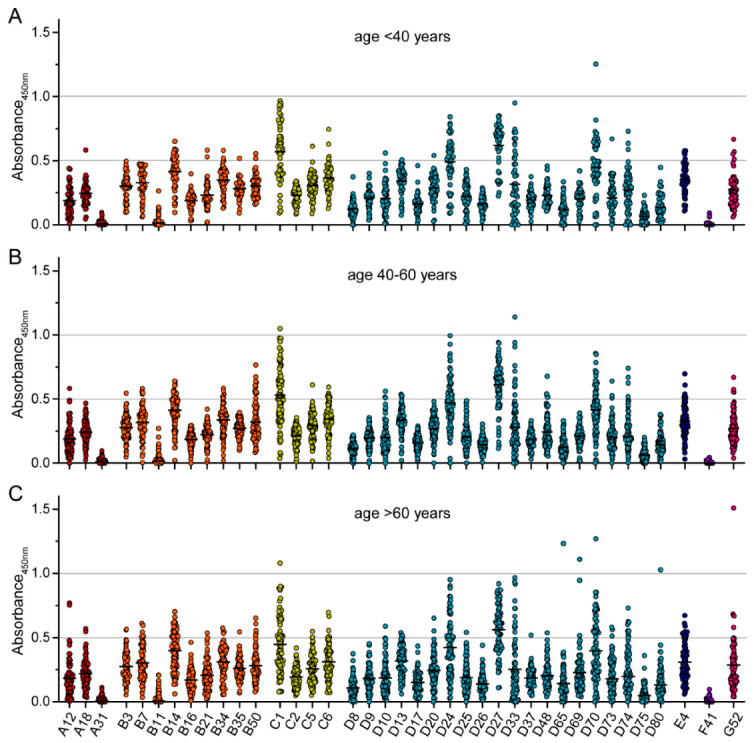
Binding antibody levels stratified by age of serum donors. The data presented in Figure 1 were stratified by the age of the serum donors and grouped as (**A**) below 40 years of age (*n* = 49), (**B**) 40 to 60 years of age (*n* = 84), and (**C**) older than 60 years (*n* = 76). Each dot represents an individual sample, lines indicate mean values of all sera for the indicated HAdV type. No statistically significant differences in binding antibody levels of the different age groups were found using Kruskal-Wallis one-way ANOVA on ranks and Dunn’s multiple comparisons test.

**Figure 4:**
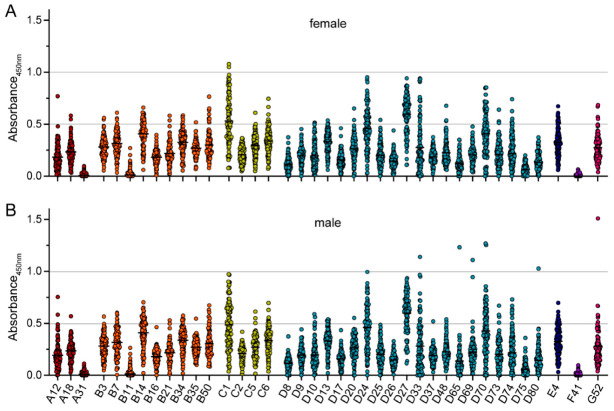
Binding antibody data stratified by sex of serum donors. The data presented in Figure 1 were stratified by the sex of the serum donors, showing data from 107 female (**A**) and 102 male serum donors (**B**). Each dot represents an individual sample, lines indicate mean values of all sera for the indicated HAdV type. No statistically significant differences in binding antibody levels of female and male serum donors were found using Kruskal-Wallis one-way ANOVA on ranks and Dunn’s multiple comparisons test.

When we stratified the binding antibody data by the age of the serum donors, no significant differences between the three age groups of younger than 40 years, 40–60 years old and older than 60 years were detected (Figure 3). Only very slight trends to lower antibody levels in the oldest group for a few viruses, such as the species C HAdV types, were observed. As expected, there were also no significant differences in the binding reactivity of sera collected from female or male donors (Figure 4).

**Figure 5:**
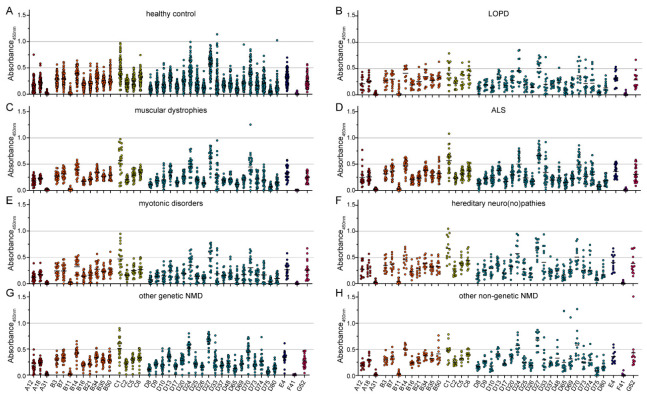
Binding antibody data stratified by health status of serum donors. The data presented in Figure 1 were stratified by the health status of the serum donors, showing data of 76 healthy serum donors (**A**), 14 serum donors with LOPD (**B**), 25 serum donors with muscular dystrophies (**C**), 26 serum donors with ALS (**D**), 20 with myotonic disorders (**E**), 13 with hereditary neuro(no)pathies (**F**), 24 with other genetic NMD (**G**) and 11 serum donors with non-genetic NMD (**H**). Each dot represents an individual sample, lines indicate mean values of all sera for the indicated HAdV type. No statistically significant differences in binding antibody levels of the different NMD groups compared to the healthy control group were found using Kruskal-Wallis one-way ANOVA on ranks and Dunn’s multiple comparisons test.

When we compared the binding antibody levels in the healthy control group and the seven NMD groups, no significant differences were found (Figure 5), even though the sera from patients with muscular dystrophies or motoneuron disease showed a trend toward slightly higher binding antibody reactivity against most HAdV types.

**Figure 6:**
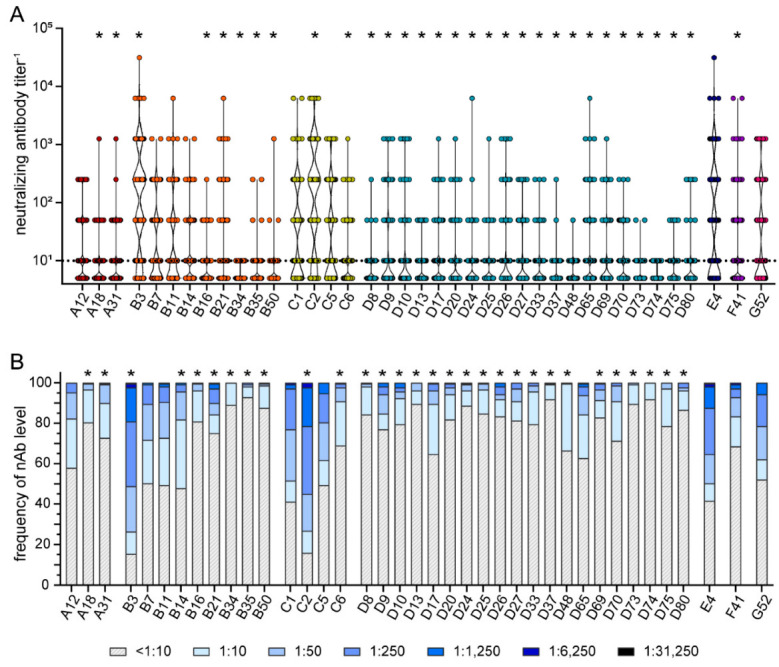
Neutralizing antibody levels and prevalence. Neutralizing antibodies were analyzed using 209 samples of healthy volunteers and volunteers with NMD. Serum samples were serially diluted and tested for their reactivity against the 39 indicated HAdV types. The highest serum dilutions that resulted in neutralization of 90% of input virus for each serum sample for the indicated HAdV types are shown in (**A**). The percentage of serum samples with the indicated neutralizing antibody levels against the indicated HAdV types are shown in (**B**). Each dot represents an individual sample. Significant differences in neutralizing antibody levels (**A**) compared to HAdV-C5 are indicated by * (*p* < 0.05, Kruskal-Wallis one-way ANOVA on ranks, Dunn´s multiple comparisons test). Significant differences in neutralizing antibody prevalence (**B**) compared to HAdV-C5 are indicated by * (*p* < 0.05, Chi square test with Bonferroni adjustment for multiple comparisons).

We next analyzed the neutralizing antibody levels in the sera and found the highest neutralizing reactivity against HAdV-B3, HAdV-C2, HAdV-E4, HAdV-C1, HAdV-G52, HAdV-C5, and HAdV-F41 (in descending order, Figure 6A). The lowest neutralizing reactivity of the sera was observed against HAdV-D74, HAdV-B34, HAdV-D73, HAdV-D37, HAdV-D48, HAdV-D13, HAdV-D75, HAdV-D8, HAdV-B35, and HAdV-B16. Compared to the neutralizing reactivity against HAdV-C5, we found significantly higher neutralization levels against HAdV-B3 and HAdV-C2. On the other hand, significantly lower neutralizing antibody levels compared to HAdV-C5 were detected against HAdV-A18 and -31, HAdV-B16, -B21, -B34, -B35, -B50, HAdV-C6, all species D HAdV types, and HAdV-F41. Similar results were obtained when we analyzed the prevalence of neutralizing antibodies in the cohort (Figure 6B). It has to be pointed out that against many species A, B, and D HAdV types, but also against HAdV-C6 and -F41, more than 80% of the sera showed no neutralizing antibody reactivity or only at a dilution of 1:10.

**Figure 7:**
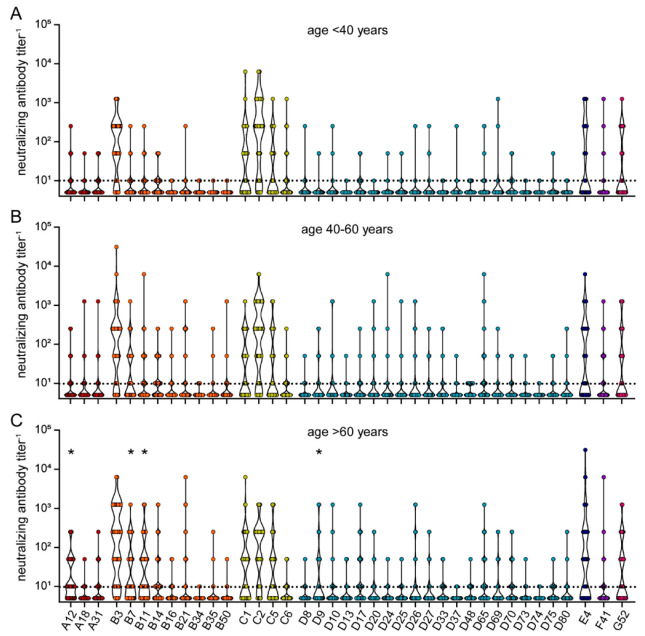
Neutralizing antibody data stratified by age of serum donors. The data presented in Figure 5 were stratified by the age of the serum donors and grouped as (**A**) below 40 years of age (*n* = 49), (**B**) 40 to 60 years of age (*n* = 84), and (**C**) older than 60 years (*n* = 76). Each dot represents an individual sample. Significant differences in neutralizing antibody levels compared to the age group “< 40 years” are indicated by * (*p* < 0.05, Kruskal-Wallis one-way ANOVA on ranks, Dunn´s multiple comparisons test).

When we stratified the neutralizing antibody data by age, sera from donors older than 60 years showed significantly higher neutralizing reactivity against HAdV-A12, HAdV-B7, HAdV-B11, and HAdV-D9 compared to sera from donors younger than 40 years (Figure 7). A trend toward higher reactivity was also observed for some other HAdV types such as HAdV-E4 and some species B and D types.

**Figure 8:**
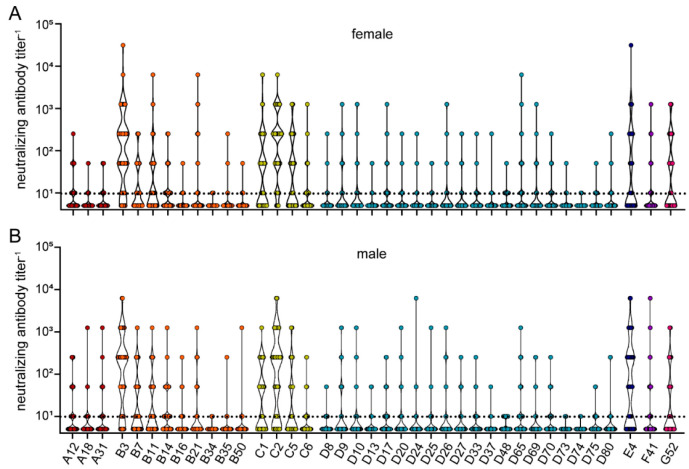
Neutralizing antibody data stratified by sex of serum donors. The data presented in Figure 5 were stratified by the sex of the serum donors, showing data from 107 female (**A**) and 102 male serum donors (**B**). Each dot represents an individual sample. No statistically significant differences in neutralizing antibody levels of female and male serum donors were found using Kruskal-Wallis one-way ANOVA on ranks and Dunn’s multiple comparisons test.

Stratification of the neutralizing antibody data by sex of the serum donors again revealed no statistically significant differences (Figure 8), confirming that the sex is not a determining factor for antibody prevalence against HAdV.

**Figure 9:**
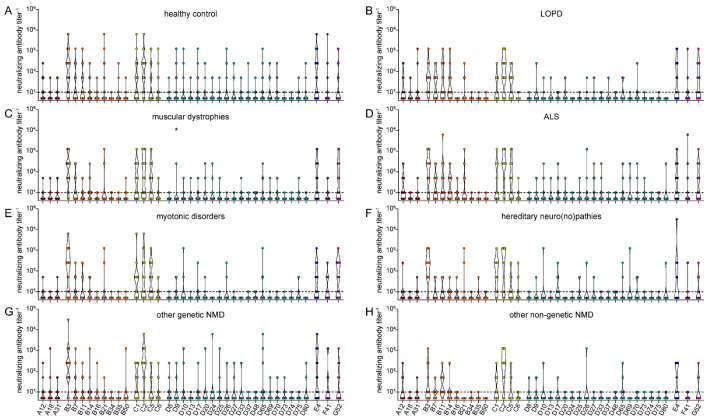
Neutralizing antibody data stratified by health status of serum donors. The data presented in Figure 5 were stratified by the health status of the serum donors, showing data from76 healthy serum donors (**A**), 14 serum donors with LOPD (**B**), 25 serum donors with muscular dystrophies (**C**), 26 serum donors with ALS (**D**), 20 with myotonic disorders (**E**), 13 with hereditary neuro(no)pathies (**F**), 24 with other genetic NMD (**G**) and 11 serum donors with non-genetic NMD (**H**). Each dot represents an individual sample. Significant differences in neutralizing antibody levels compared to the healthy control group are indicated by * (*p* < 0.05, Kruskal-Wallis one-way ANOVA on ranks, Dunn´s multiple comparisons test).

When we stratified the neutralizing antibody data according to the health status of the serum donors, we found only a minor yet statistically significantly lower level of HAdV-D9 neutralizing antibodies in the sera of patients with muscular dystrophy compared to the healthy control group, and no statistically significant differences for the other patients with NMD (Figure 9).

## 4. Discussion

Our new data presented here provide a comprehensive picture of the prevalence of both binding and neutralizing antibodies against 39 HAdV types in a cohort of adult patients with and without NMD. We were able to identify a number of HAdV types with low binding as well as low neutralizing antibody levels, such as HAdV-A31, -B16, -B34, -B35, -D8, -D37, -D48, -D73, -D74, -D75, and -D80, that may be promising candidates for further characterization and possible development as gene therapy vectors. Further characterization would include a detailed analysis of target cell tropism and immunogenicity. While a strong immunogenicity is a desirable feature of vaccine vectors, the opposite is true for gene therapy vectors. In this regard, some HAdV types such as HAdV-D48 have already been pursued as vaccine vectors and were found to be rather poorly immunogenic [58,59], which might make them preferred gene therapy vector candidates.

Similar to our previous findings using sera from young adults [54], we found a discrepancy between binding and neutralizing antibody levels for some of the viruses tested in this analysis: while the binding antibody levels against HAdV-A31, -B11, and -F41 were very low, neutralizing antibodies against these viruses were detectable in many sera, although at rather low levels for HAdV-A31. On the other hand, we found rather high levels of binding antibodies against some species D HAdV types, but only low levels of neutralizing antibodies. A regression analysis revealed no correlation between binding and neutralizing antibody levels with the exception of HAdV-C1, where a weak positive correlation was observed (Supplementary Figure S1). These findings emphasize the value of analyzing both binding and neutralizing antibody levels side by side.

It is important to note that the levels of binding and neutralizing antibodies in the sera of patients with NMD were not significantly different from those in healthy controls. Compared to our previous study of the seroprevalence in young adults [54], we observed major differences only for some of the HAdV types tested. The binding antibody levels against HAdV-A31 were very low in the current cohort compared to much higher levels found in the student cohort, whereas the opposite was true for HAdV-B50 and HAdV-C6, for which we detected rather high binding antibody levels in the NMD cohort compared to low levels detected in the student cohort. This finding implies that while these HAdV types are candidates that could be further pursued, they would not be universally applicable. Patients should be screened before receiving vectors based on these types, which would be feasible for gene therapy but less so for more widespread use as vaccine vectors. With regard to neutralizing antibodies, we found a slightly higher overall prevalence of neutralizing antibodies against all tested HAdV types, which was especially pronounced for HAdV-E4. It is interesting to note, however, that the comparison of the different age groups in the current cohort showed that the influence of age was not very pronounced, with significantly higher levels in the older age group detected only for a few viruses. It still has to be kept in mind that all participants were adults and we did not include any children in the present study, and it has been well documented that young children acquire higher antibody levels when growing older due to increasing numbers of contact with different viruses [52]. It has also been shown before that the exposure of certain age groups to specific HAdV types may lead to differences in antibody levels [52,60]. One limitation of our study is that we only included sera from patients living in Germany, and it cannot be assumed that the seroprevalence in this cohort is representative of the global prevalence. Indeed, it has been well documented that seroprevalence can vary significantly between [61,62,63,64,65] and even within countries [66]. It would therefore be highly desirable to perform similar seroprevalence studies with serum samples collected in other parts of the world. Compared to a previous study by Vogels et al. who tested serum samples from Belgian blood donors [53], we found a similar prevalence of neutralizing antibodies against species B, D and E HAdV types, but a substantially lower prevalence of neutralizing antibodies against species A HAdV types. Interestingly, while Vogels et al. demonstrated the highest prevalence for HAdV-C5 compared to the other HAdV types, the prevalence of neutralizing antibodies against HAdV-B3 and -C2 was significantly higher than that against HAdV-C5 in our cohort. Furthermore, we also found the binding antibody response to be highest against HAdV types other than HAdV-C5, which had not been analyzed in studies of other groups.

It should also be pointed out that the relative widespread use of AdV-based vaccines against SARS-CoV-2 [15,16,67,68] may have induced higher specific or cross-specific HAdV antibodies. It would be very interesting, and shall be the aim of future research, to analyze how the seroprevalence of different HAdV types has been influenced by these vaccinations.

While individual NMDs are considered rare diseases, prevalence rates between 0.05 and 20 per 100,000 population (see [69] for a meta-analysis) indicate that more than 300,000 people in Europe are affected by various NMDs. Gene replacement is an important treatment option for this group of patients, and while AAV have been developed into the first effective NMD gene therapy [19], large transgene sizes require vectors of greater capacity for gene therapy of some NMDs, such as Duchenne muscular dystrophy [30]. HAdV are excellent candidates for this application and our study reveals a range of potential candidates from HAdV species A, B, and D. 

Pre-existing immunity against the vector is an important factor for the success of gene therapy, especially when the gene therapy vectors are administered systemically. High levels of pre-existing antibodies will interfere with transduction efficacy and impair transgene delivery to the target tissues [9,70]. Some gene therapy and oncolytic virus therapy trials have specified exclusion criteria with regard to HAdV neutralizing antibodies and excluded patients with HAdV-neutralizing antibody titers ranging from 1:320 to 1:1000 [71,72,73,74]. Our data show that for some species A, B, and D HAdV types, all serum donors in our study would be below such exclusion titers, confirming these types as promising candidates that are worth pursuing. Besides pre-existing antibody responses, pre-existing HAdV-specific CD4^+^ and CD8^+^ T cell responses also have the potential to impair the efficacy of transgene delivery [75]. On the other hand, an inflammatory response against the vector is not only mediated by pre-existing adaptive immune responses, but also by innate immune responses induced by capsid components (reviewed in [76]). A severe inflammatory response can lead to major complications in patients and has been the cause of the first death in an HAdV-C5 based gene therapy trial [77]. A better understanding of pre-existing HAdV-specific T cell responses and a detailed characterization of the immunogenicity and reactogenicity of the different HAdV types will therefore also be required for an informed decision on future vector candidates.

Further research will also have to determine the transduction efficiency of muscle cells and neurons by the different HAdV types to identify the most promising candidates. The receptor usage is widely different for the different HAdV types (reviewed in [78]) and involves molecules such as the coxsackie and adenovirus receptor (CAR), the membrane cofactor protein / CD46, desmoglein, glycans, and integrins. While it has already been demonstrated that the expression levels of CAR and CD46 are low in differentiated muscle cells [79,80,81] and neurons [82], more detailed studies will have to be performed for a side-by-side analysis of the transduction efficiency of the different HAdV types.

Finally, on the practical side it should also be considered that the growth properties of the different HAdV types in cell culture are very different, as can also be seen from the widely different incubation times required for the neutralization assay [54]. A favorable propagation time would be another desirable property of an HAdV vector candidate, allowing for effective production in the future.

In conclusion, we found that patients with NMD have very similar levels of binding and neutralizing antibodies against HAdV compared to healthy individuals, and we identified HAdV-A31, -B16, -B34, -B35, -D8, -D37, -D48, -D73, -D74, -D75, and -D80 as promising candidates for further characterization and future vector development. 

## Figures and Tables

**Table 1:** Characteristics of the serum donor cohort. NMD: neuromuscular disorder, SD: standard deviation, TIA: transient ischaemic attack, d.a.: data acquired.

	Healthy	NMD Patients	Total	*p* Value *
**Serum donors**	76	133	**209**	
**Age (mean) (SD)**	60.12 (20.68)	52.15 (15.76)	**55.04 (18.12)**	0.00449
age < 40 years (n)	15	34	**49**	
age 40–60 years (n)	24	60	**84**	
age > 60 years (n)	37	39	**76**	
**Weight (kg) (SD)**	76.71 (15.59)	77.38 (19.46)	**77.14 (18.16)**	0.78906
**Height (cm) (SD)**	170.8 (12)	172.2 (10.4)	**171.8 (11.1)**	0.38357
**BMI (kg/m²) (SD)**	26.08 (4.01)	25.98 (5.58)	**26.02 (5.07)**	0.85816
d.a. on weight and height (%)	73 (96)	129 (97)	**202 (96.7)**	
**Sex**				0.07991
male (%)	31 (40.8)	71 (53.4)	**102 (48.8)**	
female (%)	45 (59.2)	62 (46.6)	**107 (51.2)**	
**Neuromuscular Disorder**				
late-onset Pompe disease (LOPD) (%)		14 (10.5)		
Muscular Dystrophies (%)		25 (18.8)		
Degenerative motoneuron disorder (ALS) (%)		26 (19.6)		
Myotonic Disorders (%) Hereditary Neuro(no)pathies (%) other genetic NMD (%) other non-genetic NMD (%)		20 (15.0) 13 (9.8) 24 (18.1) 11 (8.3)		
**other diseases**				
Hypertension (%)	33 (43.4)	37 (27.8)	**70 (33.5)**	0.02580
Diabetes mellitus (%)	4 (5.3)	11 (8.27)	**15 (7.18)**	0.39409
conditions after spinal disc herniation, spinal stenosis (%)	4 (5.3)	8 (6)	**12 (5.74)**	0.82038
conditions after infarct (all localisations), TIA (%)	35 (46.1)	5 (3.76)	**40 (19.14)**	<0.00001
Hypothyroidism (%)	4 (5.3)	17 (12.8)	**21 (10.05)**	0.05435
Depression (%)	3 (3.95)	6 (4.51)	**9 (4.31)**	0.84523
**Smoking (%)**	12 (19.05)	30 (24.79)	**42 (22.83)**	0.22559
d.a. on smoking (%)	63 (82.89)	121 (91)	**184 (88.04)**	

* Statistically significant differences between the groups were tested with unpaired, two-tailed *t* test.

## Data Availability

The data presented in this study are available on request from the corresponding author.

## Supplementary Materials

The following supporting information can be downloaded at: https://www.mdpi.com/article/10.3390/v15010079/s1, Figure S1: Correlation analysis of binding and neutralizing antibody levels.
