# Supplementary material for: Seroprevalence of Binding and Neutralizing Antibodies against 39 Human Adenovirus Types in Patients with Neuromuscular Disorders
Source: Viruses. 2022 Dec 27;15(1):79. doi: 10.3390/v15010079 (PMC9866721; doi:10.3390/v15010079)
Supplement: Supplementary file 1 [file viruses-15-00079-s001.zip › viruses-2100121-supplementary.pdf]

Supplementary Figure S1

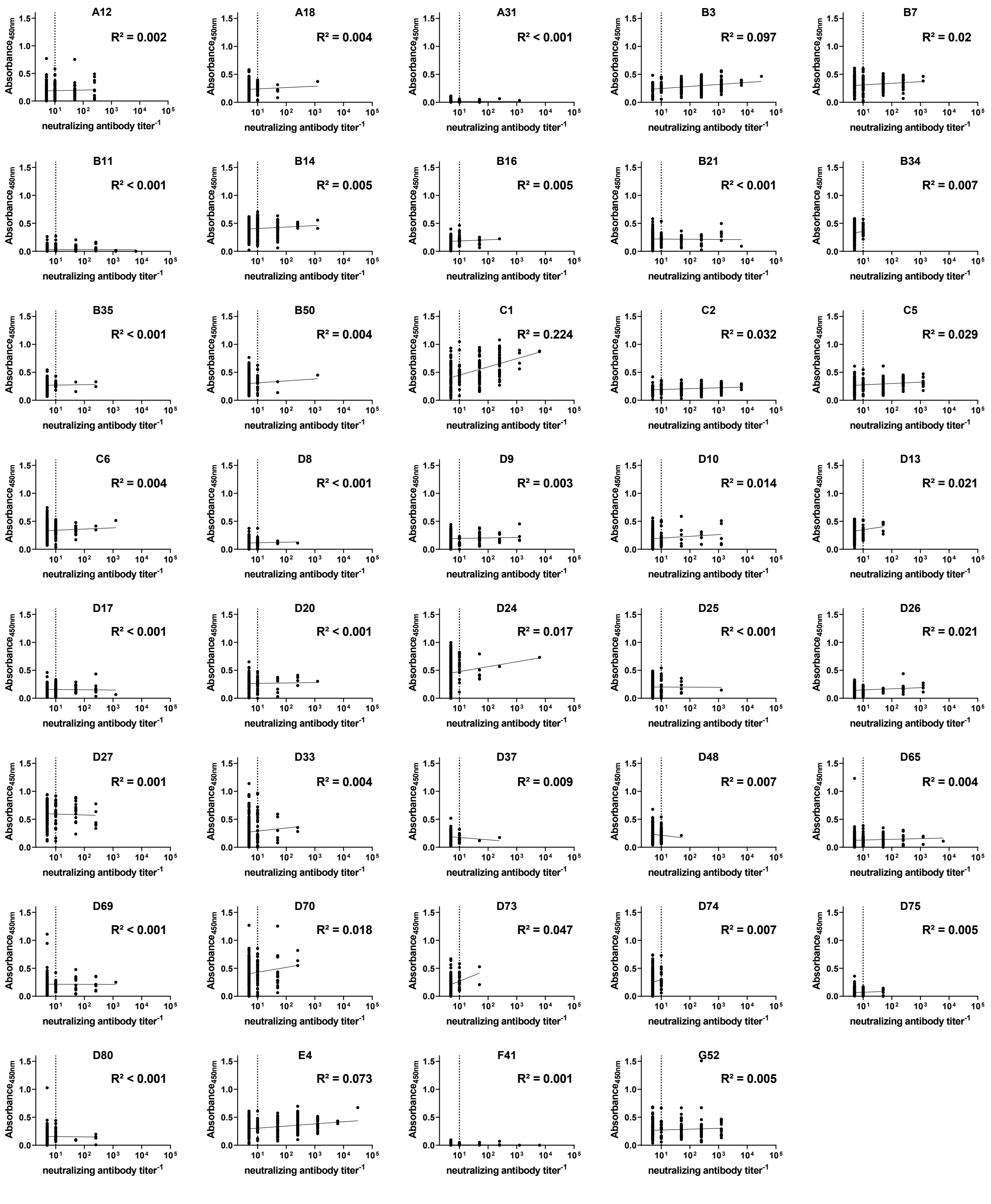

Correlation analysis of binding and neutralizing antibody levels.

Data for binding and neutralizing antibody levels from 209 sera against the indicated HAdV types were subjected to least squares regression analysis. No correlation between binding and neutralizing antibody levels was observed for most HAdV types, with the exception of HAdV-C1, where a weak positive correlation was detected.
